# Supplementary material for: Identification of photoperiod-induced specific miRNAs in the adrenal glands of Sunite sheep (Ovis aries)
Source: Front Vet Sci. 2022 Jul 22;9:888207. doi: 10.3389/fvets.2022.888207 (PMC9354845; doi:10.3389/fvets.2022.888207)
Supplement: Supplementary file 1 [file Data_Sheet_1.ZIP › Supplementary file/miRDeep2 file.zip!/miRDeep2 file/Know_Structure/SP42A3/oar-mir-376e.pdf]

miRBase precursor : oar-mir-376e
Total read count : 901
oar-miR-376e-5p read count : 159
oar-miR-376e-3p read count : 583
oar-miR-376b-5p read count : 159
remaining reads : 0

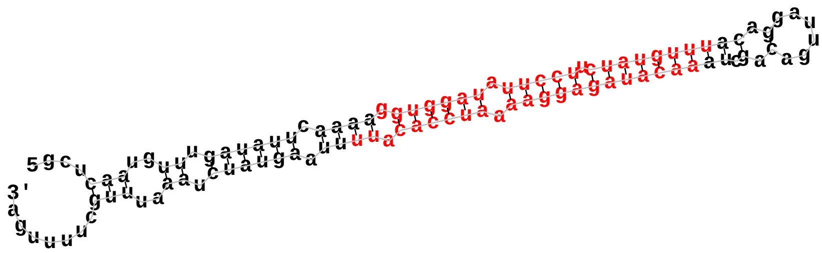

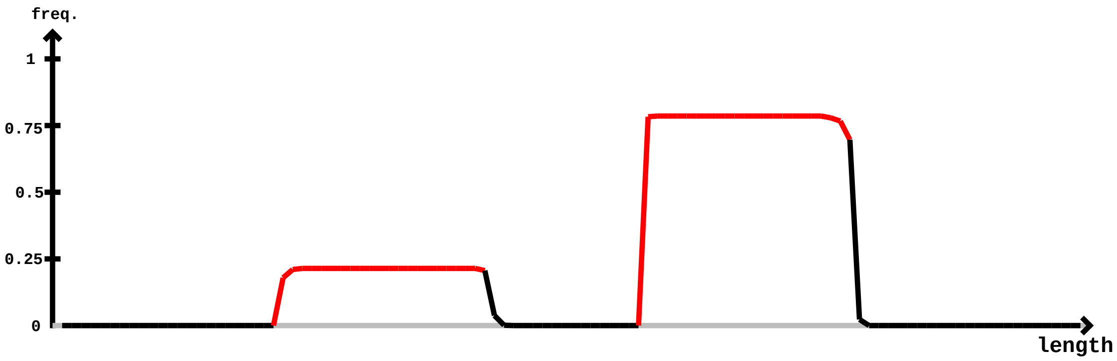


```
                                                            oar-miR-376e-3p
                        oar-miR-376b-5p
                        oar-miR-376e-5p
5'- gcucaauguuugauauucaaaagguggauauuccuucuauguuuacaggauugacagcuaaacauagaggaaaauccacauuuuaaguaucuaaauuugcuuuuga   -3'   exp
    ((.....((((((((((.((((.((((((.(((((.((((((((((.(......).).)))))))))))))).)))))).)))).)))))).))))..))......   reads   mm   sample
    ......................gguggauauuccuucuauguu...............................................................   6       0    seq
    ......................gguggauauuccuucuauguuu..............................................................   110     0    seq
    ......................gguggauauuccuucuauguuua.............................................................   17      0    seq
    .......................guggauauuccuucuauguuu..............................................................   15      0    seq
    .......................guggauauuccuucuauguuua.............................................................   8       0    seq
    ........................uggauauuccuucuauguuua.............................................................   2       0    seq
    ........................uggauauuccuucuauguuuac............................................................   1       0    seq
    ............................................................aacauagaggaaaauccac...........................   5       0    seq
    ............................................................aacauagaggaaaauccaca..........................   9       0    seq
    ............................................................aacauagaggaaaauccacau.........................   52      0    seq
    ............................................................aacauagaggaaaauccacauu........................   499     0    seq
    ............................................................aacauagaggaaaauccacauuu.......................   16      0    seq
    .............................................................acauagaggaaaauccacauu........................   1       0    seq
    .............................................................acauagaggaaaauccacauuu.......................   1       0    seq
```
